# A forensic genetic investigation reveals a captive origin for a wild alien population of raccoons in Italy

**DOI:** 10.1038/s41598-024-62424-1

**Published:** 2024-05-28

**Authors:** Luisa Garofalo, Nadia Cappai, Marco Mencucci, Emiliano Mori, Lorenzo Attili, Rita Lorenzini

**Affiliations:** 1 Istituto Zooprofilattico Sperimentale del Lazio e della Toscana “M. Aleandri”, Roma, Italy; 2 Parco Nazionale delle Foreste Casentinesi, Monte Falterona e Campigna, Pratovecchio, Italy; 3 Reparto Carabinieri Parco “Foreste Casentinesi”, Pratovecchio, Italy; 4 Istituto di Ricerca sugli Ecosistemi Terrestri IRET, Consiglio Nazionale delle Ricerche, Sesto Fiorentino, Italy; 5 National Biodiversity Future Center, Palermo, Italy; 6 Centro di Referenza Nazionale per la Medicina Forense Veterinaria, Istituto Zooprofilattico Sperimentale del Lazio e della Toscana “M. Aleandri”, Grosseto, Italy

**Keywords:** Genetics, Zoology, Invasive species

## Abstract

Invasive alien species have extensively impacted the ecosystems, where they may affect the native biodiversity. The North American raccoon *Procyon lotor* is one of the most successful invaders in Europe since its introduction in the early twentieth century. In Italy, a wild population was first established in the North at the beginning of the 2000s following a local introduction event. A further self-sustaining population was reported ten years later in Central Italy. To support an official investigation by the authorities, who suspected a captive origin of the free-ranging raccoons in Central Italy, we used nuclear and mitochondrial DNA markers, combined with different statistical approaches, to characterise their gene pool and trace the source of the founders. Results revealed that founders came from a private zoo-park from which they had inadvertently escaped, soon establishing a reproductive population in the wild. Additionally, our mitochondrial DNA data were used to supplement the haplotype variability known to date in captive and wild raccoons from Europe, Asia and their native range. The comparisons allowed us to update previous networks based on the control region with a new mitochondrial lineage, which had not been detected so far.

## Introduction

Biological invasions represent one of the most important causes of the ongoing Sixth Global Biodiversity crisis, being responsible for over one-fifth of species extinctions since 1500^1^. Particularly, species directly or indirectly introduced by humans into areas where they were not historically present may affect native taxa and ecosystems by means of competition, predation, disease/parasitic transmission and hybridisation^2,3^. The annual rate of first records worldwide has increased during the last 200 years, with 37% of all first records reported in the last four decades^4^, when the number of invasive alien species has dramatically increased particularly in Europe, with extensive impacts on the native biodiversity^5^. Escapes from captivity are one of the main pathways of alien species introduction^6^, often resulting in self-sustaining populations^7–10^. Since 2014, the European Union Regulation 1143/2014 has been adopted to prevent and manage the introduction and spread of invasive taxa, based on a list of alien species of Union concern, identified through detailed risk assessments^11^. The Regulation includes a ban on import, trade, breeding and release into the environment of the listed species.

Amongst mammals, the Northern raccoon *Procyon lotor* (hereafter, raccoon) is present in this priority list. This carnivoran is native to Central America, USA and Canada (where it is considered a pest), and has been introduced to Europe since the early twentieth century for the fur farms, as a pet, and as an attraction in urban zoo-parks^12^. Unintentional escapes triggered the invasion process in Europe, where established and expanding populations of raccoon occur in more than twenty countries^13,14^ (Global Biodiversity Information Facility-GBIF at https://www.gbif.org/species/5218786/).

In Italy, first records of the raccoon in the wild date back to 2004 in Lombardy, where a local introduction event led to a reproductive population along the Adda river^15–17^. Afterwards, several single records have been reported for other areas, including Northern (Piedmont, Trentino-South Tyrol, Emilia-Romagna, Liguria) and Central (Marches, Tuscany, Latium) regions^16^ (records on www.inaturalist.org). In 2020, the zoonotic parasitic nematode *Baylisascaris procyonis* was described for the first time in free-ranging raccoons from the Casentino valley, Tuscany, with a prevalence of 41.9%^18,19^, emphasising the risks to human health (with possible fatal outcomes of the infections) caused by the introduction of alien species and calling for urgent interventions.

In Central Italy, it all began in 2013, when a raccoon was found dead inside the Foreste Casentinesi, Monte Falterona e Campigna National Park (FCNP), in the Casentino valley^20^. Later, three live raccoons were camera-trapped inside and outside the FCNP. In 2015, retrieval of a second raccoon carcass led the FCNP officers to undertake an investigation to establish the origin for the invaders and try to stem their spread in the valley. A private zoo-park, located nearby the FCNP borders and close to the first raccoon found dead, was suspected to be the source from which some captive raccoons had inadvertently escaped. In the absence of a specific law against the intentional spread of alien species in nature, the FCNP reported the zoo-park owner to the authorities for “abandonment of animals”, a crime provided for by art. 727 of the Italian penal code. Meanwhile, during the years 2015–2019, the distribution and numbers of raccoons in Central Italy had increased, and a self-sustaining population had clearly arisen^21^. Therefore, a legal eradication plan to stop the invasion was necessarily implemented by the FCNP.

As part of the fact-finding process, the law enforcement officers instructed our laboratory to carry out genetic analyses to verify that the free-ranging raccoons in the Casentino valley came from the investigated zoo-park, with the ultimate aim of attributing responsibility for their escape to the owner. To do that, we analysed the free-ranging raccoons, the captive raccoons hosted in the private zoo-park, and, for comparison with unrelated individuals, raccoons sampled from several rescue centres and zoos in Europe, presumably coming from different areas of their native range. We also collected samples of raccoons from Lombardy, where the only wild population dwelt so far in Italy^16^.

We combined data from mitochondrial (mt) and nuclear biparental DNA markers with the following specific aims: 1. Compare the mtDNA lineages of free-ranging raccoons from the Casentino valley for matching with captive individuals from the investigated zoo-park; 2. Identify membership of the haplotypes obtained in the free-ranging raccoons to haplogroups already known for raccoons introduced to Europe or, alternatively, reveal new mitochondrial lineage(s); 3. Verify that the free-ranging raccoons shared kinship relationships with raccoons from the zoo-park, while sharing no kinship at all with the other sampled raccoons. The DNA evidence was intended as support to the prosecution's theory about “abandonment of animals” in a trial against the defendant. Additionally, our genetic results are discussed in the framework of raccoon invasion in Europe.

## Results

### Mitochondrial CR sequencing

Sequencing of mitochondrial partial Control Region (CR) from 75 raccoon samples produced seven haplotypes covering a 550-base-pair (bp) long segment (Table 1), differing at 25 variable sites (24 transitions and one transversion). Three haplotypes (PLO13, PLO2a, PLO2b) were previously described by Frantz et al.^22^ in wild raccoons introduced to Germany and later found in populations from Belgium and France^23^. Two haplotypes (PLO32a, PLO57a) matched the shorter (467 bp) haplotypes PLO32 and PLO57 found in natural populations from North-Eastern America and in captive raccoons from Germany and France^23–25^. By contrast, haplotypes Lotor1 and Lotor2 were first identified in this study and had never been described before either in wild or captive/introduced raccoons. Lotor1, Lotor2, PLO32a, PLO57a have been registered in GenBank (https://www.ncbi.nlm.nih.gov/genbank/) with accession numbers OP341729-OP341732.

**Table 1:** List of raccoon samples.

ID	Sampling site	Status	N	Tissue	mtCR
CV_I	Casentino Valley (Arezzo, Italy)	W	15	muscle/blood	PLO2a
LO_I	Lombardy (Italy)	W	3	muscle	PLO13
ZP_I	Zoo-park (Arezzo, Italy)	C	5	saliva	PLO2a
MA_I	Monte Adone (Bologna, Italy)	C	5	hair	PLO13
SE_I	Semproniano (Grosseto, Italy)	C	1	muscle	PLO2a
VC_I	Valcorba (Padova, Italy)	C	2	hair	PLO2a
CE_I	Cecina (Livorno, Italy)	C	5	hair	PLO13
SL_I	Safari Langhe (Cuneo, Italy)	C	5	hair	PLO13
NE_G	Neuwied (Germany)	C	3	hair	PLO2a, PLO57a
SP_G	Springe (Germany)	C	5	hair	PLO2a, PLO13
HE_G	Heidelberg (Germany)	C	5	hair	PLO2a
SA_F	Saint Aignan (France)	C	5	hair	**Lotor2**
AN_B	Antwerp (Belgium)	C	11	hair	PLO2a, PLO13, **PLO32a**, **PLO57a**, **Lotor1**
FU_S	Fuerteventura (Spain)	C	5	hair	PLO2b, PLO13

W, wild; C, captive; N, sample size; mtCR, mitochondrial control region haplotype (550 bp). Haplotype nomenclature follows Cullingham et al.^24^ and Frantz et al.^22^. New haplotypes from this study are in bold.

**Figure 1:**
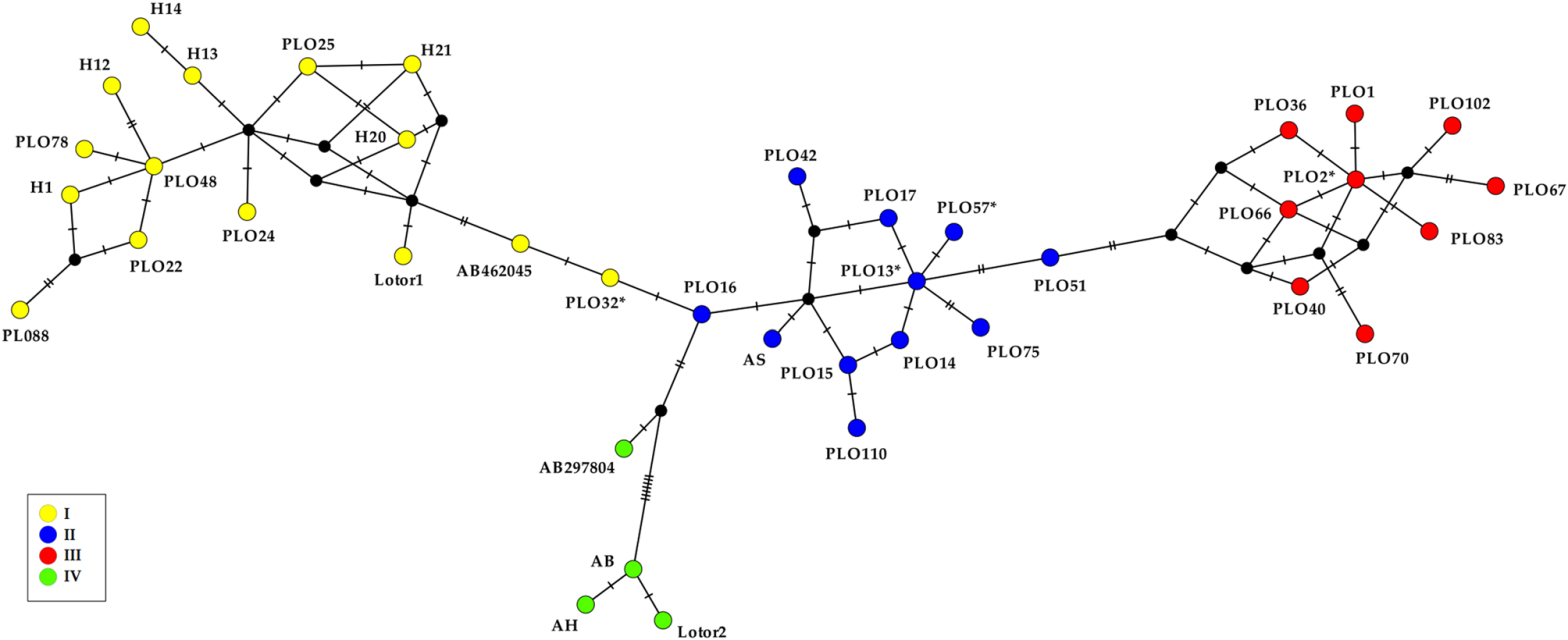
Median joining network based on the alignment (467 bp) of 39 mitochondrial CR sequences obtained in this study and from the literature. Bar-lines represent single nucleotide mutations, while black dots indicate unsampled haplotypes. Sequences tagged “PLO” were first identified by Cullingham et al.^24^; the ones indicated with accession numbers AB462045 and AB297804 are from Tokutomi et al. (direct submission on 27 Sept 2008) and Takada et al. (direct submission on 12 Mar 2007); AH, AB, AS are from Okuyama et al.^26^; Lotor1 and Lotor2 are new sequences from this survey. * = haplotypes corresponding to our longer sequences (550 bp). Haplotype PLO2* includes PLO2a, Plo2b (550 bp)^22^ and PLO2c (mitogenome AB291073, Yonezawa et al. direct submission on 22 Jan 2007). Colours identify mitochondrial lineages.

A median joining network (Fig. 1) was derived based on a trimmed 467-long alignment to include a set of shorter sequences available online, comprising the most frequent haplotypes carried by raccoons introduced to Europe so far. The network showed three already known mitochondrial lineages (I, II, III), in accordance with Cullingham et al.^24^ and Frantz et al.^22^, and, additionally, a fourth, highly distant new lineage (IV) which encompassed three haplotypes bore by raccoons introduced to Japan (AB297804, AB, AH)^26^ and one (Lotor2) of our new haplotypes, found in captive raccoons from France. A minimum of eight point mutations separated this fourth lineage from PLO16 (see below). Haplotype Lotor1 was found in raccoons from captivity in Belgium and joined lineage I. In the network, haplotype PLO16 appeared as a connecting sequence among three lineages (I, II, IV), in contrast to the networks obtained by Cullingham et al.^24^ and Frantz et al.^22^, where it joined lineage II. Following these authors, however, we also tagged PLO16 as belonging to lineage II (Fig. 1). Raccoons from the free-living population in the Casentino valley, as well as the captive raccoons from the private zoo-park under investigation, all showed haplotype PLO2a, which belonged to lineage III. On the contrary, raccoons from the only other wild population in Italy (Lombardy) bore haplotype PLO13, clustering instead into lineage II.

### STR genotyping

All 11 STR loci were highly polymorphic, showing from 6 (PLM01) to 14 (PLO-M15) alleles per locus in the entire dataset. As expected from a highly substructured sample with putative inbreeding between many individuals, three loci (PLOT-02, PLM13, PLO-M15) showed deviation from Hardy–Weinberg (HW) expectations (*p* < 0.001), while 19 out of 55 pairwise comparisons (34%) between loci across the whole dataset deviated significantly from linkage equilibrium (*p* < 0.001). However, it should be emphasised that, while violations of assumptions for linkage and HW equilibria due to small sample sizes and inbreeding may lower the power of downstream analyses, the presence of clear-cut relationships among the genotypes of close relatives can still be identifiable^27^. Consequently, for our purposes, no locus was excluded from downstream analyses.

**Figure 2:**
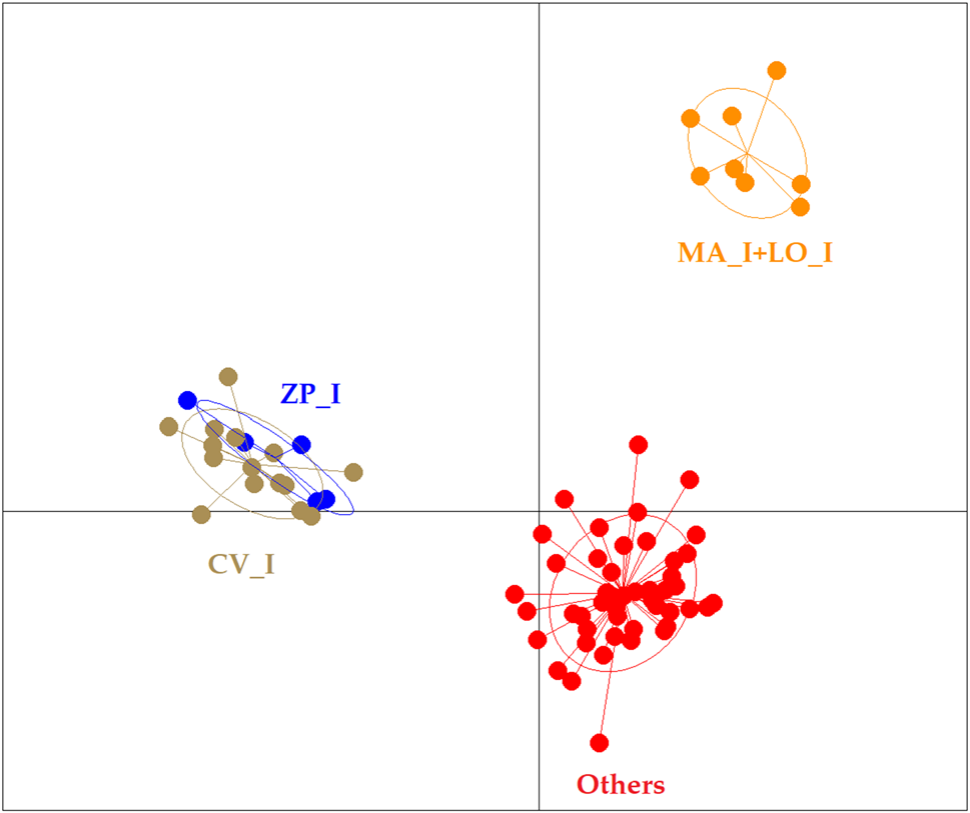
DAPC scatterplot based on 11 STR loci describing the genetic variability in the sampled raccoons, represented by first (X-axis) and second (Y-axis) principal components. The graph features single individuals as dots and groups as inertia ellipses. IDs for sampling sites as in Table 1.

The genetic variability in our dataset was qualitatively described using a Discriminant Analysis of Principal Components (DAPC, Fig. 2). Cross-validation procedure suggested that five principal components (PC) were retained in the analysis as a proxy for optimal description of the original data. The first two PCs best summarised most of the allele diversity, and clearly defined three clusters of genetically close individuals. The first PC identified a separate cluster which included the free-living raccoons from the Casentino valley (CV_I) and the captive individuals held in the private zoo-park (ZP_I). The second PC separated a cluster encompassing raccoons of the wild population in Lombardy (LO_I) and captive animals kept in a rescue centre in Monte Adone, Bologna (MA_I). All the other raccoons clustered into a single separate group of genotypes. When the most divergent cluster (CV_I + ZP_I) was excluded from the analysis, the captive raccoons from Monte Adone still closely joined the wild raccoons from Lombardy, and their cluster always remained clearly separated (data not shown). When analysing only individuals of the “Others” cluster, no structure was apparent anymore (data not shown). A STRUCTURE Bayesian analysis was conducted to assess the probability of ancestry for each multilocus genotype in one (or more) genetic clusters (K) ranging from 2 to 13. The Puechmaille’s method identified K = 9 as the most probable value for the number of inferred clusters (Supplementary Fig. S2a). In accordance with the DAPC, the CV_I and ZP_I, as well as the LO_I and MA_I raccoons, were probabilistically assigned to two private clusters respectively, while the remaining raccoons fell into alternative clusters or showed admixed ancestry in more than one cluster (Supplementary Fig. S2b). By running STRUCTURE without CV_I, ZP_I, LO_I and MA_I raccoons, no more private clusters emerged (data not shown).

The probability of identity for siblings (PID_sib_) in the entire dataset was 7.07 × 10^–5^, meaning that approximately 0.07 raccoons in a sample of 1000 individuals (PID_sib_/1000) could show, by chance, the same multilocus genotype. When wild and captive raccoons from the Casentino valley were considered as a cluster of genetically close individuals, as suggested by the DAPC and STRUCTURE analyses, then PID_sib_/1000 dropped to one order of magnitude (8.40 × 10^–6^), which is indicative of high relatedness between individuals.

**Figure 3:**
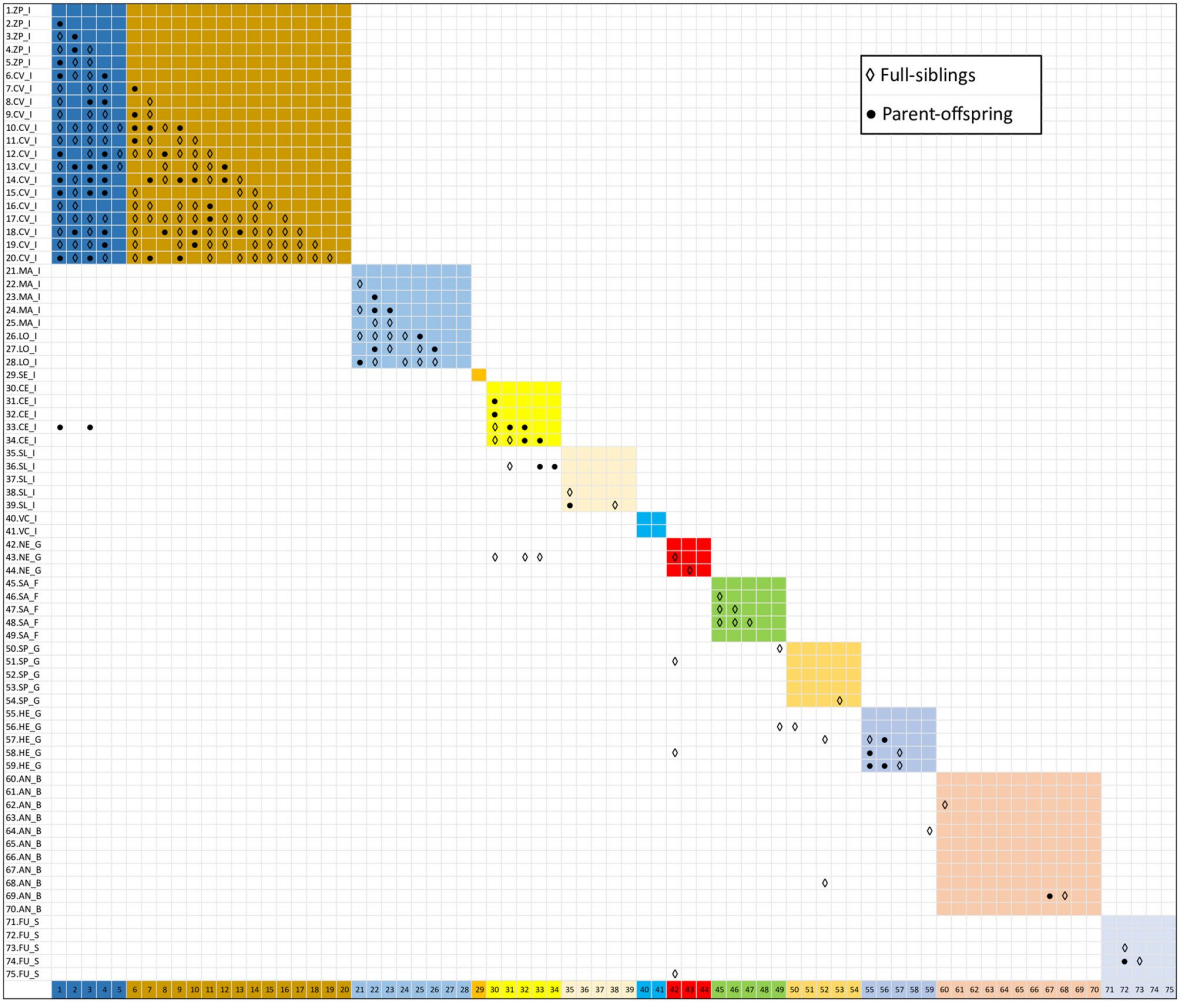
Matrix of results from ML-RELATE. Pairs of related individuals were identified as full-siblings and parent-offspring. Unrelated or not closely related dyads (i.e. half-sibs) are not tagged. Coloured squares encompass individuals from the same captive colony/wild population. IDs for sampling sites as in Table 1.

To find pedigree relationships between individuals using ML-RELATE, maximum likelihoods for full-sibling (FS) and parent-offspring (PO) dyads were estimated and compared to unrelated as an alternative hypothesis. In order to obtain conservative values, which corresponds to deriving a minimum number of related pairs^28^, null alleles were not accounted for in the calculations. Wild raccoons from the Casentino valley (CV_I) and captive raccoons from the zoo-park (ZP_I) were very closely related (Fig. 3), showing 23% parent–offspring dyads (44 out of 190 pairwise comparisons), and 54% full-siblings (103 out of 190 pairwise comparisons). Only 23% dyads were unrelated or less closely related (say, half-sibs). No genetic relationships were found between CV_I raccoons and raccoons from other provenances in Italy (either captive or wild) and Central Europe, with the exception of two significant PO relationships with two individuals held in a rescue centre in Cecina, Italy (CE_I). Although not documented, an exchange of animals between colonies in captivity can reasonably account for this result. Close relatedness was also found between wild raccoons from Lombardy and captive individuals held in the Monte Adone rescue centre, as also suggested by the DAPC scatterplot. This clearly indicates a common origin, likely due to the shelter in this centre of animals caught in the wild. High levels of inbreeding were also detected within the colony in Cecina, where all except one pairwise comparison revealed FS or PO relationships. Same situation was found in the captive raccoons from Heidelberg, Germany, where only three out of ten comparisons showed not closely related genotypes.

Basically, these results were confirmed by the analyses using the software Colony and Cervus. While relying on a group-likelihood approach instead of a pairwise likelihood method, Colony identified with high levels of probability (0.70 < *p* < 0.90) all except nine of the 103 full siblings inferred by ML-RELATE in the captive and wild raccoons from the Casentino valley. Regarding the parent–offspring pedigrees, each PO dyad, as suggested by ML-RELATE in the CV_I and ZP_I raccoons, was confirmed by Cervus with strict confidence at 95%. Moreover, the most probable parent was identified from a shortlist of plausible (i.e. non-excluded) candidates which all belonged to the Casentino valley group. Except for two PO relationships involving raccoons from the rescue centre in Cecina (see above), for no other PO pair has a candidate parent been identified from elsewhere other than the Casentino valley, confirming the absence of plausible PO relationships between wild raccoons from Central Italy and the other raccoons in the sample set.

## Discussion

The impact exerted by invasive alien species—i.e. exotic species which may cause environmental or economic damages, or affect human health and wellness—require proactive strategies to halt their invasion, as well as a focus on prevention^29^. Therefore, determining the origins of naturalised invasive species is pivotal to understand pathways of invasions and possibly, to prevent further releases. The goal, however, is not straightforward. As for the raccoon, one of the world's most successful invaders, to date it had never been possible to establish the captive origin of founders introduced throughout Europe. This applies, for example, to Germany, where raccoons were introduced as early as the 1930s^13^, but also to Belgium, France, Luxemburg and Spain. In these countries, the number of introduction/escape events has been genetically traced, but neither the captive sources nor the geographic origins in the native range has been identified for any of the introduced raccoon populations^22,23,25,30,31^. In this paper, we report a genetic investigation based on nuclear and mitochondrial DNA evidence that, for the first time and beyond a reasonable doubt, identified the captive source of a self-sustaining population of raccoons in the wild.

Until the first decade of the 2000s, the only wild population of raccoons in Italy occurred in Lombardy, Northern Italy. Ten years later, a further small breeding population was reported in the Casentino valley, Central Italy. Following this observation, a neighbouring national park, fearing a massive invasion, lodged with the authorities an official complaint. As part of the official enquiry that ensued, we showed through genetic evidence that the source of the free-roaming raccoons was a nearby private zoo-park from which the animals had escaped. In particular, the use of highly informative STR markers revealed close kinship of the wild-caught raccoons from the Casentino valley to all individuals hosted in the investigated private zoo-park, while comparatively no kinship was shown either with captive raccoons sampled across Europe or with wild raccoons in Italy. Our 11-loci panel proved to be reliable and effective in differentiating raccoon gene pools and resolving kinship relationships, even when highly inbred individuals were involved and different statistic approaches were used. For example, if the population was dramatically large and inbred (e.g. counting more than 15,000 sibling raccoons), our set of STR loci would still be capable to discriminate single individuals and genetically reconstruct genealogies. Moreover, since relatedness analyses were performed under a very conservative approach, with false negatives that were preferred to untrue parent/offspring and full-sibling relationships (so that, in the view of a trial, the defense attorney's hypothesis was favoured), our conclusions appear to be highly supported.

Data from mtDNA analysis corroborated the results from nuclear biparental markers. Wild and captive raccoons of the Casentino valley shared the same and unique maternal CR haplotype PLO2a, which pleads for a common origin. It should be mentioned, however, that PLO2, together with PLO13, is one of the most frequent haplotypes found both in native raccoons from North America and in raccoons introduced worldwide^22–25^, which suggests poor diagnostic capability for this marker. That said, however, haplotype PLO2 was absent from wild raccoons of Lombardy, thereby excluding this population as source for raccoons of the Casentino valley (see also below).

Beyond the scientific side, which covers the ecosystem management in a broad sense, our genetic results would have contributed substantially in the judicial context, where they would have been probative to convict the defendant at trial. Nevertheless, they were not, because the owner of the zoo-park died during the laboratory analyses, thereby the prosecution was discontinued and the crime was settled.

The cue from the court case allowed us to collect data on mtDNA variation and include our results in the framework of the genetic lineages found in raccoons outside their native range. Most of the haplotypes obtained in our entire samples set were already known in captive and wild raccoons throughout Europe^22,23,25,31^. Additionally, we found two new haplotypes, Lotor1 and Lotor2, in raccoons from zoos in Belgium and France, respectively. Incidentally, while writing this paper, haplotype Lotor2 was also found in one individual kept illegally in a private garden in Northern Italy, from which it escaped but was promptly caught by the forestry officers. This haplotype, in particular, belonged to a new and very distant haplogroup (IV in our Fig. 1) that had never been identified in the networks or trees published so far, in which only three main lineages had consistently emerged^22–25,31^. One explanation for this might be that the haplotypes forming this new lineage (AB, AH, AB297804) were either not considered in previous phylogenetic analyses, or were included in haplogroups II-III^32^. Consequently, the lineage was left undetected. These overlooked haplotypes were previously found in individuals introduced into Japan, where raccoons were entered in the 1970s as pets and fur animals, and soon spread as feral throughout Hokkaido^26^ and the Boso Peninsula^32^. Apart from this emerging haplogroup IV, the number and phylogenetic relationships of the remaining haplogroups in our network are fully in line with earlier studies.

Noteworthy, the free-ranging raccoons from the Casentino valley and those from the population in Lombardy bore different and highly divergent haplotypes, PLO2a and PLO13, joining lineages III and I, respectively. This pleads for two independent colonisation events for the wild raccoons in Italy, as also suggested by the STR-based DAPC and STRUCTURE analyses. We are aware that our small sample from the Northern Italian population calls for caution in this conclusion, as larger sampling might reveal other haplotypes besides PLO13. However, since this population originated most probably from a small number of founders following a single escape event^16^, it likely bears reduced mitochondrial variability. Additionally, the finding of the roundworm *Baylisascaris procyonis* in the raccoons of the Casentino valley, but not in those from Lombardy^33^ is another clue to their different origins.

## Conclusion

The invasion by alien species is becoming a plague worldwide. The raccoon is potentially one on the most problematic invaders both to ecosystems and human health and, at the same time, one of the most successful alien species, due to high adaptability, mobility, reproductive rate, and virtually lack of natural predators in the newly colonised habitats. For these reasons, eradication strategies implemented anywhere outside the species’ native range have rarely been completely effective^13^. In some of the raccoon-invaded areas, genetic investigations, through the analysis of mitochondrial and nuclear markers, have provided important information on the number of anthropogenic introductions, but they have never managed to trace the captive source from which self-sustaining populations originated. Here, for the first time, use of genetic markers and different statistical approaches allowed us to identify a private zoo-park as the source for the founders of one of the two wild populations currently established in Italy. Genetic-based information on the origin, either intentional or accidental, of free-ranging raccoons is crucial to keep their spreading (at least) under control, if not really to succeed in their complete eradication. In Italy, for example, wild raccoons have been subjected to eradication plans since recent decades on biological and public health grounds. Nevertheless, the number of reports of free-ranging individuals outside these two known areas of occurrence is alarmingly increasing, and suggests that, despite the efforts, raccoon invasion still remains an ongoing and unresolved issue.

## Methods

### Ethical approval

No animals were captured and/or sacrificed specifically for the purposes of this study, nor were any laboratory experiments or investigations conducted on live animals. Most of the carcasses used in this study were obtained following legal eradication plans in accordance with a state-instituted eradication programs aimed at removing alien species. Additional carcasses were obtained from road accidents. For these reasons, no ethics permit in accordance with the EU Directives was required for this study. Non-invasive samples (hairs, saliva) were collected from live captive raccoons that were handled in accordance with ARRIVE guidelines. Saliva samples of live raccoons from a private zoo-park were collected with the consent of the owner by a veterinarian officially appointed by the Italian authorities in the course of an investigation on crime against animals. According to Directive 2010/63/EU, Article 1 point 5 (http://data.europa.eu/eli/dir/2010/63/2019-06-26), defining which practices are considered non-experimental, and Commission Regulation (EC) N. 865/2006, Articles 54 and 55 (https://eur-lex.europa.eu/eli/reg/2006/865/oj), laying down detailed rules concerning individuals of wild species in captivity, no ethics permit was required for collecting saliva samples, and no animal research ethics approval from the ethics committee was needed to use the samples either in the molecular tests linked to the investigation process, nor in the present study.

### Samples

Salivary samples were collected from live captive raccoons hosted in the investigated private zoo-park (ZP_I, Table 1, Supplementary Figure S1). Hairs were gathered from raccoons in rescue centres and zoos in Italy and Central Europe. Muscle and blood samples were obtained from raccoons removed from the wild under legal eradication plans in the Casentino valley, and opportunistically from animals that died accidentally throughout the course of this study. A total of 75 raccoon samples were analysed for mitochondrial and nuclear diversity.

### DNA extraction and laboratory analyses

DNA was isolated from approximately 15 mg of muscle, 5–10 hairs, and salivary swabs using the QIAamp DNA Mini Kit (QIAGEN, Hilden, Germany) and the Maxwell16 Instrument (Promega, Madison, USA) for automated genomic DNA isolation, and diluted in 200 µl RNAse-free molecular grade water. DNA quantification was performed using the Quantus Fluorometer (Promega) according to the manufacturer’s instructions. Extracted DNAs were stored at + 4 °C until amplification steps. One mock tube with reagents and no template DNA was included in each extraction session. A fragment of approximately 600 bp in the mtDNA CR was amplified and sequenced using the raccoon-specific primers PLO-L15997 and PLO-CRL1^22,24^. PCR reactions contained 2.5 μl of 10X Gold buffer (Thermo Fisher Scientific, Waltham, MA, USA), 200 μM of each dNTP, 2.5 mM MgCl_2_, 10 pm of each primer, 1U of AmpliTaqGold polymerase (Thermo Fisher Scientific), 3 µl template DNA (10–50 ng) and PCR grade H_2_O in a final volume of 25 μl. PCR tubes were loaded onto an ABI Veriti® 96-Well Thermal Cycler (Thermo Fisher Scientific) under the following thermal cycling conditions: an initial 5 min denaturation step at 95 °C, 35 cycles of 30 s at 95 °C, 30 s at the annealing temperature (56 °C), 2 min extension step at 72 °C, followed by 5 min at 72 °C. PCR products were cleaned up with the QIAquick PCR purification kit (QIAGEN) and sequenced bidirectionally using the BigDye Terminator kit v3.1 (Thermo Fisher Scientific) with the same primers used in the amplification. Unincorporated dyes and other contaminants were removed with the Agencourt® CleanSEQ solution (Beckman Coulter, Beverly, MA, USA), then sequences were loaded on an ABI Prism™ 3130 Genetic Analyzer (Thermo Fisher Scientific), and analysed using the Sequencing Analysis Software v5.3.1 (Thermo Fisher Scientific).

Multilocus genotypes of raccoons were obtained through co-amplification of 11 selected nuclear STR loci^34–36^ optimised for two multiplexes: M1 (PLOT-02, PLOT-05, PLM09, PLM13, PLO-M15, PLM17) and M2 (PLOT-10, PLM01, PLM03, PLM06, PLM14). Each multiplex contained 3 µl of genomic DNA, 10 pm of each primer, 3.6 µl of master mix (QIAGEN Multiplex PCR Kit, code 206,143) and PCR grade H_2_O in 12 µl total reaction volume. Multiplex amplifications consisted of an initial 15 min activation step at 95 °C, followed by 35 cycles of denaturation at 94 °C for 30 s, annealing at 58 °C for 90 s, extension at 72 °C for 60 s, and a final extension step at 60 °C for 30 min. Extraction negative controls and PCR mock tubes were included in each amplification round. A volume of 2 μl of five-fold diluted amplified reaction products was denaturated with 14 μl HiDi™ formamide and combined with 0.2 μl GeneScan™ 500 LIZ™ size standard (Thermo Fisher Scientific) for fragment sizing on an ABI Prism™ 3130 Genetic Analyzer. GeneMapper Software 5.0 (Thermo Fisher Scientific) was used for fragment sizing and allele calling.

### Data analysis

#### Mitochondrial DNA

Mitochondrial CR sequences were aligned and edited using the multiple alignment program included in the package Vector NTI v9.1 (Invitrogen, Carlsbad, CA, USA) and AliView^37^. In order to compare the obtained sequences to those available in the literature and find mtDNA haplogroup membership for our raccoon samples at a larger scale, the original alignment (550 bp) was trimmed to cover a shorter common fragment of 467 bp in length (accession numbers of our and online sequences are listed in Supplementary Table S1). A median-joining network based on final 39 sequences was constructed using PopArt^38^.

#### Nuclear STRs

We obtained individual STR profiles with our 11-marker panel and performed a Discriminant Analysis of Principal Components, DAPC^39^, using the Adegenet package 2.1.6^40^ for the R software 4.2.1 on the web interface. This multivariate approach allowed us to visualise and qualitatively describe the genetic variation in our dataset and to identify clusters of genetically close individuals, without relying on any underlying evolutionary model. A Bayesian assignment procedure as implemented in the software STRUCTURE v.2.3.4^41^ was used to complement the multivariate analysis and to identify clusters (K) of genetically close individuals under HW assumptions (without any prior non-genetic information) and an admixture model based on correlated allele frequencies^42^. Ten repetitions for each K ranging from 2 to 13 were performed in a Markov Chain of Monte Carlo with 10 × 10^6^ iterations and 10 × 10^5^ steps of burn-in. The Puechmaille’s method^43^, as performed in StructureSelector^44^ on the web (http://Imme.ac.cn7StructureSelector/), was used to assess the most likely number of meaningful genetic clusters while reducing the impact of uneven sample size across groups.

In order to assess the reliability of our 11-locus panel for the identification of single raccoons, the probability that two individuals drawn randomly from a population will exhibit identical multilocus genotypes by chance (P_ID_) was derived from the STR dataset. P_ID_ estimates depend on the genetic variability and the number of loci analysed, as well as on the presence of close relatives in the study population(s). As many of our samples came from captive nuclei of likely inbred raccoons, to cope with this issue, we used Gimlet^45^ to derive estimates of P_ID_ among siblings (P_IDsib_), which was suggested as a highly conservative upper boundary for individual identification^46^.

Relatedness and ancestry between the multilocus genotypes of our raccoons were inferred using ML-RELATE^47^. This program calculates the maximum likelihood estimates of full-sibling (FS) and parent-offspring (PO) pedigrees for each pair of individuals in the whole dataset. Pedigrees are tested against the alternative hypothesis of unrelated relationship, and the most plausible genealogy is found between two individuals, which is consistent with the observed data. Statistical significance was established via simulation, according to Kalinowski et al.^47^.

Parentage and sibship inference were further explored with Cervus v3.0.7^48^ and Colony v2.0.6.8^49^, respectively. In Cervus, a pairwise likelihood-based method was developed to assign offspring to the most likely mother (and/or father) within a set of non-excluded candidate parents, at known levels of statistical confidence through simulations. Alternatively, Colony is based on a full-pedigree likelihood approach, rather than on single pairwise comparisons (dyads), meaning that the likelihood for full-siblings is considered over the entire pedigree configuration, which generally increases significantly the accuracy of inference^49^. Although all three software are able to account for genotyping errors at single loci (null alleles, dropouts, mistyping), we deliberately assigned an error rate of zero in the calculations, in order not to favour false exclusions and to obtain highly conservative values. In other words, given the alleged high level of inbreeding in the raccoons from CV_I and ZP_I, false negatives were preferred to untrue PO and FS relationships. HW deviations and linkage equilibrium of markers were tested with Genepop v4.7.5 on the web (https://genepop.curtin.edu.au/)^50^.

### Supplementary Information

Supplementary Information.

## Data Availability

New mitochondrial haplotypes have been deposited in GenBank with accession numbers OP341729- OP341732.

## References

[1] Bellard C, Cassey P, Blackburn TM (2016). Alien species as a driver of recent extinctions. Biol. Lett..

[2] Mack RN (2000). Biotic invasions: Causes, epidemiology, global consequences, and control. Ecol. Appl..

[3] Kumschick S (2015). Ecological impacts of alien species: quantification, scope, caveats, and recommendations. BioScience.

[4] Seebens H (2017). No saturation in the accumulation of alien species worldwide. Nat. Commun..

[5] Turbelin AJ, Malamud BD, Francis RA (2017). Mapping the global state of invasive alien species: Patterns of invasion and policy responses. Glob. Ecol. Biogeogr..

[6] Hulme PE (2009). Trade, transport and trouble: Managing invasive species pathways in an era of globalization. J. Appl. Ecol..

[7] Cassey P, Hogg CJ (2015). Escaping captivity: The biological invasion risk from vertebrate species in zoos. Biol. Conserv..

[8] Ancillotto L, Strubbe D, Menchetti M, Mori E (2016). An overlooked invader? Ecological niche, invasion success and range dynamics of the Alexandrine parakeet in the invaded range. Biol. Invasions.

[9] Cucco M (2021). The spreading of the invasive sacred ibis in Italy. Sci. Rep..

[10] Gargioni C, Monaco A, Francesco Ficetola G, Lazzeri L, Mori E (2021). From the Andes to the Apennines: Rise and fall of a free-ranging population of feral llamas. Animals.

[11] Roy HE (2018). Developing a framework of minimum standards for the risk assessment of alien species. J. Appl. Ecol..

[12] Salgado I (2018). Is the raccoon (*Procyon lotor*) out of control in Europe?. Biodivers. Conserv..

[13] Stope MB (2023). The raccoon (*Procyon lotor*) as a neozoon in Europe. Animals.

[14] Kochmann J, Cunze S, Klimpel S (2021). Climatic niche comparison of raccoons *Procyon lotor* and raccoon dogs *Nyctereutes procyonoides* in their native and non-native ranges. Mammal Rev..

[15] Canova L, Rossi S (2009). First records of the northern raccoon *Procyon lotor* in Italy. Hystrix Ital. J. Mammal..

[16] Mori E (2015). The masked invader strikes again: The conquest of Italy by the Northern raccoon. Hystrix Ital. J. Mammal.

[17] Mazzamuto MV (2020). When management meets science: adaptive analysis for the optimization of the eradication of the Northern raccoon (*Procyon lotor*). Biol. Invasions.

[18] Lombardo A (2022). First report of the zoonotic nematode *Baylisascaris procyonis* in non-native raccoons (*Procyon lotor*) from Italy. Parasit. Vectors.

[19] Lombardo A (2023). Detection of endoparasites in non-native raccoons from central Italy. Vet. Sci..

[20] Cappai, N. *et al.* Alien species: Raccoon (*Procyon lotor*) in Foreste Casentinesi National Park. *X Congresso Italiano di Teriologia Acquapendente (VT)* (2016).

[21] Boscherini A, Mazza G, Menchetti M, Laurenzi A, Mori E (2019). Time is running out! Rapid range expansion of the invasive northern raccoon in central Italy. Mammalia.

[22] Frantz AC (2013). Limited mitochondrial DNA diversity is indicative of a small number of founders of the German raccoon (*Procyon lotor*) population. Eur. J. Wildl. Res..

[23] Larroque J (2023). Microsatellites and mitochondrial evidence of multiple introductions of the invasive raccoon *Procyon lotor* in France. Biol. Invasions.

[24] Cullingham CI, Kyle CJ, Pond BA, White BN (2008). Genetic structure of raccoons in eastern North America based on mtDNA: Implications for subspecies designation and rabies disease dynamics. Can. J. Zool..

[25] Fischer ML (2017). Multiple founder effects are followed by range expansion and admixture during the invasion process of the raccoon (*Procyon lotor*) in Europe. Divers. Distrib..

[26] Okuyama MW (2020). Genetic population structure of invasive raccoons (*Procyon lotor*) in Hokkaido, Japan: Unique phenomenon caused by pet escape or abandonment. Sci. Rep..

[27] Wang J (2016). Individual identification from genetic marker data: Developments and accuracy comparisons of methods. Mol. Ecol. Resour..

[28] Morrissey MB, Wilson AJ (2005). The potential costs of accounting for genotypic errors in molecular parentage analyses. Mol. Ecol..

[29] Simpson A (2009). Invasive species information networks: Collaboration at multiple scales for prevention, early detection, and rapid response to invasive alien species. Biodiversity.

[30] Fischer ML (2015). Historical invasion records can be misleading: Genetic evidence for multiple introductions of invasive raccoons (*Procyon lotor*) in Germany. PloS One.

[31] Alda F (2013). Genetic evidence for multiple introduction events of raccoons (*Procyon lotor*) in Spain. Biol. Invasions.

[32] Hirose M, Yoshida K, Inoue E, Hasegawa M (2021). Population genetic structure of raccoons as a consequence of multiple introductions and range expansion in the Boso Peninsula. Japan. Sci. Rep..

[33] Romeo C (2021). Lost and found: Helminths infecting invasive raccoons introduced to Italy. Parasitol. Int..

[34] Fike JA, Drauch AM, Beasley JC, Dharmarajan G, Rhodes OE (2007). Development of 14 multiplexed microsatellite loci for raccoons *Procyon lotor*. Mol. Ecol. Notes.

[35] Siripunkaw C (2008). Isolation and characterization of polymorphic microsatellite loci in the raccoon (*Procyon lotor*). Mol. Ecol. Resour..

[36] Cullingham CI, Kyle CJ, White BN (2006). Isolation, characterization and multiplex genotyping of raccoon tetranucleotide microsatellite loci. Mol. Ecol. Notes.

[37] Larsson A (2014). AliView: A fast and lightweight alignment viewer and editor for large datasets. Bioinformatics.

[38] Leigh JW, Bryant D (2015). POPART: Full-feature software for haplotype network construction. Methods Ecol. Evol..

[39] Jombart T, Devillard S, Balloux F (2010). Discriminant analysis of principal components: A new method for the analysis of genetically structured populations. BMC Genet..

[40] Jombart T (2008). adegenet: A R package for the multivariate analysis of genetic markers. Bioinformatics.

[41] Pritchard JK, Stephens M, Donnelly P (2000). Inference of population structure using multilocus genotype data. Genetics.

[42] Porras-Hurtado L (2013). An overview of STRUCTURE: Applications, parameter settings, and supporting software. Front. Genet..

[43] Puechmaille SJ (2016). The program structure does not reliably recover the correct population structure when sampling is uneven: Subsampling and new estimators alleviate the problem. Mol. Ecol. Resour..

[44] Li Y-L, Liu J-X (2018). StructureSelector: A web-based software to select and visualize the optimal number of clusters using multiple methods. Mol. Ecol. Resour..

[45] Valière N (2002). GIMLET: A computer program for analysing genetic individual identification data. Mol. Ecol. Notes.

[46] Waits LP, Luikart G, Taberlet P (2001). Estimating the probability of identity among genotypes in natural populations: Cautions and guidelines. Mol. Ecol..

[47] Kalinowski ST, Wagner AP, Taper ML (2006). ML-Relate: A computer program for maximum likelihood estimation of relatedness and relationship. Mol. Ecol. Notes.

[48] Kalinowski ST, Taper ML, Marshall TC (2007). Revising how the computer program CERVUS accommodates genotyping error increases success in paternity assignment. Mol. Ecol..

[49] Jones OR, Wang J (2010). COLONY: A program for parentage and sibship inference from multilocus genotype data. Mol. Ecol. Resour..

[50] Rousset F (2008). genepop’007: A complete re-implementation of the genepop software for Windows and Linux. Mol. Ecol. Resour..

