# Supplementary material for: A forensic genetic investigation reveals a captive origin for a wild alien population of raccoons in Italy
Source: Sci Rep. 2024 May 28;14:12246. doi: 10.1038/s41598-024-62424-1 (PMC11133336; doi:10.1038/s41598-024-62424-1)
Supplement: Supplementary file 1 — Supplementary Information. [file 41598_2024_62424_MOESM1_ESM.pdf]

Supplementary Information

A forensic genetic investigation reveals a captive origin for a wild alien population of raccoons in Italy

Luisa Garofalo[1], Nadia Cappai[2], Marco Mencucci[3], Emiliano Mori[4,5], Lorenzo Attili[6], Rita Lorenzini[6]

1. Istituto Zooprofilattico Sperimentale del Lazio e della Toscana “M. Aleandri”, Roma, Italy

2. Parco Nazionale delle Foreste Casentinesi, Monte Falterona e Campigna, Pratovecchio, Italy

3. Reparto Carabinieri Parco “Foreste Casentinesi”, Pratovecchio, Italy

4. Istituto di Ricerca sugli Ecosistemi Terrestri IRET, Consiglio Nazionale delle Ricerche, Sesto Fiorentino, Italy

5. National Biodiversity Future Center, Palermo, Italy

6. Istituto Zooprofilattico Sperimentale del Lazio e della Toscana "M. Aleandri", Centro di Referenza Nazionale per la Medicina Forense Veterinaria, Grosseto, Italy

*correspondence: lorenzo.attili@izslt.it

Figure S1. Sampling sites of wild and captive raccoons in the Casentino valley, Central Italy.

Map created by the authors using QGIS 3.22 software (https://qgis.org/en/site/).

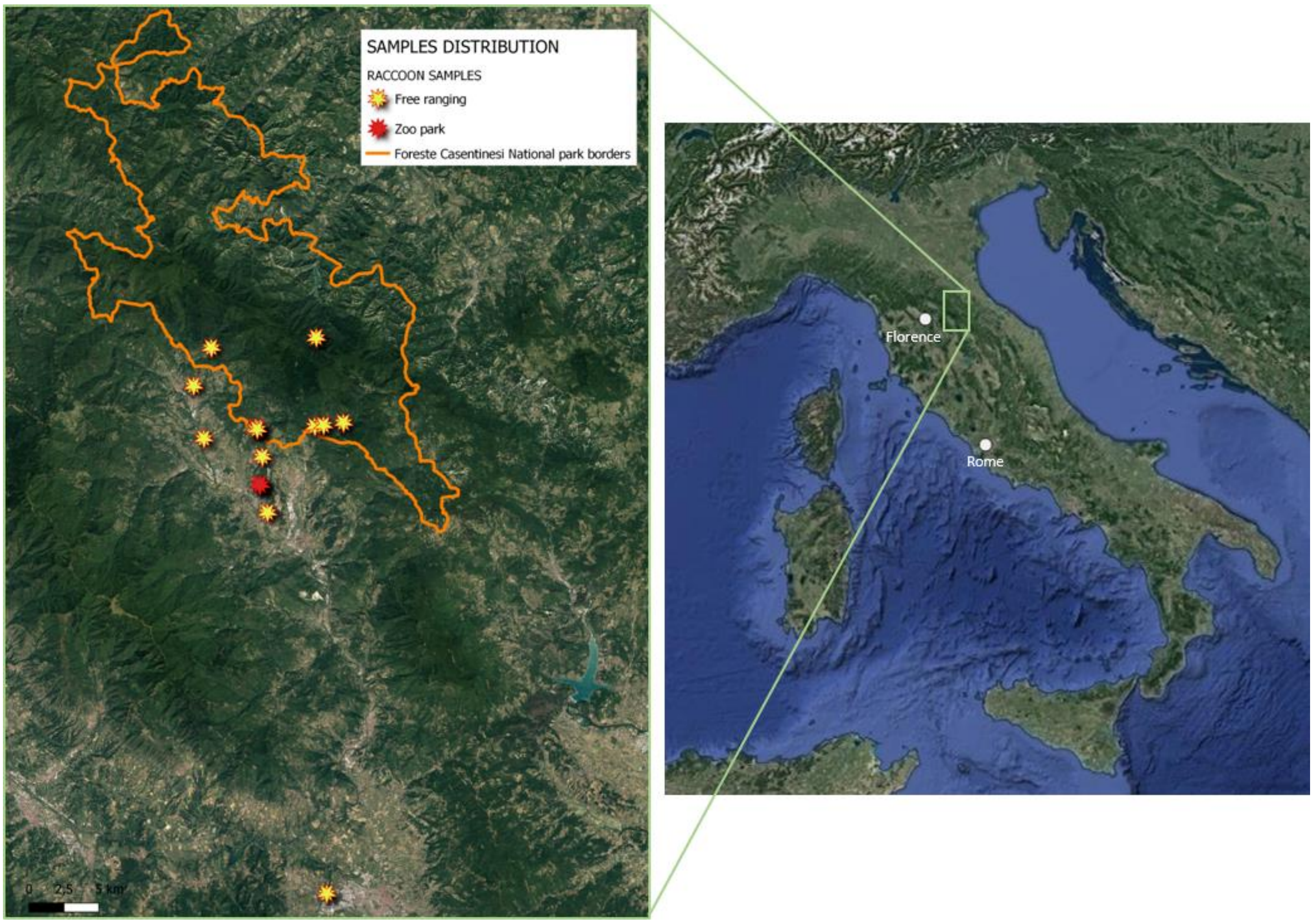

Figure S2. a) Results from Puechmaille's MedMean method, indicating K = 9 as the most probable number of genetic clusters. b) Bar plotting of the results from a Bayesian analysis conducted using STRUCTURE with K = 9. Colours indicate genetic clusters; each vertical bar represents one individual and the length of the different coloured sections is proportional to its membership in the inferred cluster(s). Raccoons from CV_I and ZP_I, as well as those from LO_I and MA_I, were grouped into two private clusters (red and yellow, respectively). All other raccoons fell into alternative clusters or showed admixed ancestry in more than one cluster. CV_I = Casentino Valley (Arezzo, Italy), LO_I = Lombardy (Italy), ZP_I = Zoo-park (Arezzo, Italy), MA_I = Monte Adone (Bologna, Italy), SE_I = Semproniano (Grosseto, Italy), VC_I = Valcorba (Padova, Italy), CE_I = Cecina (Livorno, Italy), SL_I = Safari Langhe (Cuneo, Italy), NE_G = Neuwied (Germany), SP_G = Springe (Germany), HE_G = Heidelberg (Germany), SA_F = Saint Aignan (France), AN_B = Antwerp (Belgium), FU_S = Fuerteventura (Spain).

a)

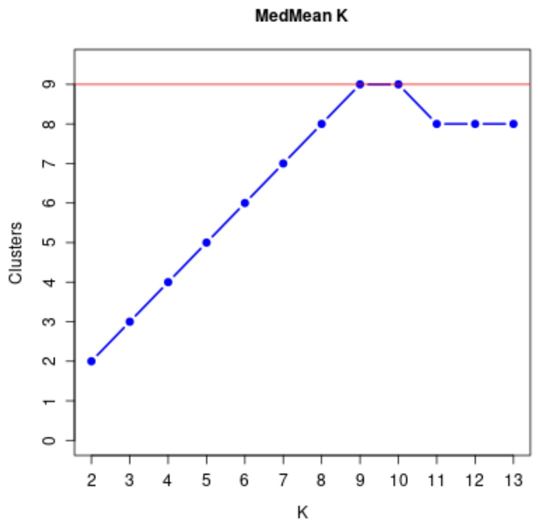

b)

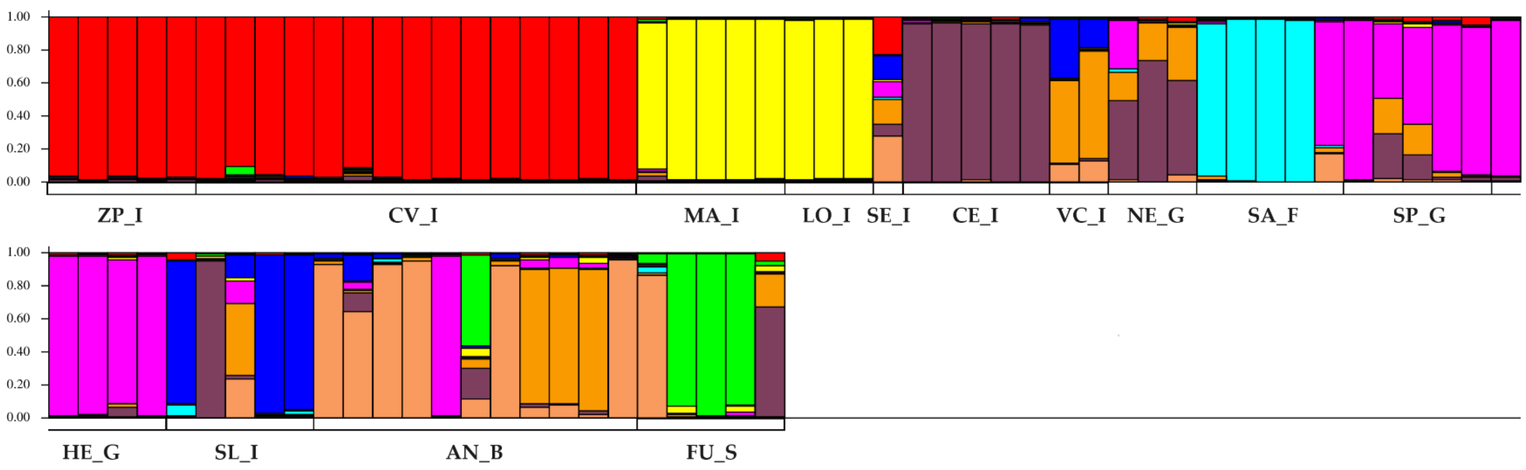

Table S1. List of mitochondrial control region sequences obtained in the present study and downloaded from GenBank, used to construct a median joining network based on a 467 bp-long alignment. * = haplotypes corresponding to our longer sequences (550 bp).

Haplotype	Accession number	Reference
Lotor1	OP341729	This study
Lotor2	OP341730	This study
PLO1	EF030392	Cullingham et al. (2008)
PLO2*	EF030393<br>KC846064 (2a)<br>KC846065 (2b)	Cullingham et al. (2008)<br>Frantz et al. (2013)<br>Frantz et al. (2013)
PLO13*	EF030345<br>KC846066	Cullingham et al. (2008)<br>Frantz et al. (2013)
PLO14	EF030346	Cullingham et al. (2008)
PLO15	EF030347	Cullingham et al. (2008)
PLO16	KC846067	Frantz et al. (2013)
PLO17	EF030349	Cullingham et al. (2008)
PLO22	EF030351	Cullingham et al. (2008)
PLO24	EF030353	Cullingham et al. (2008)
PLO25	EF030354	Cullingham et al. (2008)
PLO32*	EF030359<br>OP341731 (32a)	Cullingham et al. (2008)<br>This study
PLO36	EF030402	Cullingham et al. (2008)
PLO40	EF030404	Cullingham et al. (2008)
PLO42	EF030361	Cullingham et al. (2008)
PLO51	EF030368	Cullingham et al. (2008)
PLO57*	EF030370<br>OP341732 (57a)	Cullingham et al. (2008)<br>This study
PLO58	EF517150	Cullingham et al. (2008)
PLO66	EF030409	Cullingham et al. (2008)

PLO67	EF030410	Cullingham et al. (2008)
PLO70	EF030411	Cullingham et al. (2008)
PLO75	KC846068	Frantz et al. (2013)
PLO78	EF030378	Cullingham et al. (2008)
PLO83	EF030415	Cullingham et al. (2008)
PLO88	EF030384	Cullingham et al. (2008)
PLO102	EF030416	Cullingham et al. (2008)
PLO110	KC846069	Frantz et al. (2013)
AB	LC455752	Okuyama et al. (2020)
AH	LC455751	Okuyama et al. (2020)
AS	LC455753	Okuyama et al. (2020)
AB297804	NC009126	Takada et al. (2007) – Direct submission
AB462045	AB462045	Tokutomi & Takada (2008) – Direct submission
H1	KX357306	Trujillo and Hoffman (2017)
H12	KX357317	Trujillo and Hoffman (2017)
H13	KX357318	Trujillo and Hoffman (2017)
H14	KX357319	Trujillo and Hoffman (2017)
H20	KX357325	Trujillo and Hoffman (2017)
H21	KX357326	Trujillo and Hoffman (2017)

References

Cullingham, C. I., Kyle, C. J., Pond, B. A. & White, B. N. Genetic structure of raccoons in eastern North America based on mtDNA: implications for subspecies designation and rabies disease dynamics. *Can. J. Zool.* **86**, 947–958 (2008).

Frantz, A. C. *et al.* Limited mitochondrial DNA diversity is indicative of a small number of founders of the German raccoon (*Procyon lotor*) population. *Eur. J. Wildl. Res.* **59**, 665–674 (2013).

Okuyama, M. W. *et al.* Genetic population structure of invasive raccoons (*Procyon lotor*) in Hokkaido, Japan: Unique phenomenon caused by pet escape or abandonment. *Sci. Rep.* **10**, 8108 (2020).

Trujillo, A. L. & Hoffman, E. A. Uncovering discordance between taxonomy and evolutionary history in Florida raccoons. *Syst. Biodivers.* **15**, 74–85 (2017).
